# A Human Rights Approach to Emergency Response? The Advocacy of Canada's Human Rights Commissions during the COVID-19 Crisis

**DOI:** 10.1017/S0008423920000438

**Published:** 2020-05-06

**Authors:** Nicole De Silva

**Affiliations:** Department of Political Science, Concordia University, 1455 de Maisonneuve Boulevard West, Montreal, Quebec H3G 1M8

## Abstract

Emergencies can threaten human rights by disrupting societies, increasing vulnerabilities, and instigating exceptional measures from governments and other actors. As independent institutions mandated to protect and promote the human rights embedded in Canada's federal, provincial and territorial legislation, Canada's human rights commissions (HRCs) have mobilized to advocate for human rights during the COVID-19 public health emergency.

## Introduction

Emergencies can threaten human rights by disrupting societies, increasing vulnerabilities, and instigating exceptional measures from governments and other actors. As independent institutions mandated to protect and promote the human rights embedded in Canada's federal, provincial and territorial legislation, Canada's human rights commissions (HRCs) have mobilized to advocate for human rights during the COVID-19 public health emergency.

Based on an original dataset and content analysis of HRCs’ official statements regarding COVID-19, this research note examines the points of consistency and variation in HRCs’ advocacy for human rights during the COVID-19 crisis. This represents the first systematic analysis of advocacy by Canada's HRCs. HRCs have elucidated key human rights issues, particularly various forms of discrimination, in the context of COVID-19. HRCs also have advocated for governments and other actors (such as employers, and housing and service providers) to adopt policies and practices during the crisis that address individuals’ vulnerabilities and promote human rights. In a crisis generally framed in terms of public health and safety, comparatively analyzing Canadian HRCs’ assessments of human rights issues and obligations provides a foundation for deliberating whether and how a human rights approach to COVID-19 response should be pursued.

## Canada's Human Rights Laws and Commissions during the COVID-19 Crisis

Canada has a long history of struggling to uphold human rights during emergencies. Human rights violations under the War Measures Act during the World Wars and the 1970 October Crisis (Lindsay, 2014) contributed to the development of the Emergencies Act (1988), which explicitly recognizes the Charter of Rights and Freedoms (1982). More recently, government officials have acknowledged that COVID-19 containment and mitigation measures can and will undermine rights. Canada's Health Minister warned that defying self-isolation orders could prompt further restrictions that “put our civil liberties in jeopardy” (Georgieva, 2020), and Quebec's Director of Public Health noted COVID-19 response measures “will be violating the rights of individuals … for the collective good” (Authier, 2020).

Discussion of human rights and COVID-19 in Canada has centred on the Charter (see, for example, Macfarlane, 2020), which applies to governments and is enforceable through courts. Canada's complex system of human rights protections, however, also includes federal, provincial and territorial legislation in areas relevant to those jurisdictions (such as employment, housing and public services). There is remarkable uniformity in these laws focused on equality and antidiscrimination (Clement, 2012: 762). They all prohibit discrimination based on age, race, colour, disability, sex, sexual orientation, gender identity, and place of origin/nationality; most also cover religion, family status, gender expression, political beliefs, and sexual harassment.

This federal, provincial and territorial legislation also delegates authority to HRCs to administer and enforce human rights laws.^1^ There are a total of 13 HRCs (Table 1), and their mandates include both human rights protection and promotion (Eliadis, 2014: 35). Beyond processing human rights complaints, HRCs broadly advocate for human rights through research, policy development and public education. Not limiting their advocacy to the laws they oversee (Eliadis, 2014: 41), HRCs have increased rights consciousness and demands for wider rights (Howe and Johnson, 2000: 35) and have shaped public policy (Nierbobisz et al., 2008).

**Table 1:** Number of statements by each human rights commission

Human rights commission	Statements
Canadian Human Rights Commission (CHRC)	4
Alberta Human Rights Commission (AHRC)	1
British Columbia Office of the Human Rights Commissioner (BCOHRC)	3
Manitoba Human Rights Commission (MHRC)	3
New Brunswick Human Rights Commission (NBHRC)	1
Newfoundland and Labrador Human Rights Commission (NLHRC)	1
Northwest Territories Human Rights Commission (NTHRC)	1
Nova Scotia Human Rights Commission (NSHRC)	2
Ontario Human Rights Commission (OHRC)	6
Prince Edward Island Human Rights Commission (PEIHRC)	1
(Quebec) Commission des droits de la personne et des droits de la jeunesse (CDPDJ)	9
Saskatchewan Human Rights Commission (SHRC)	1
Yukon Human Rights Commission (YHRC)	1
Total	34

Many actors have advocated for human rights in Canada during the COVID-19 crisis, but HRCs’ advocacy is distinctly authoritative, considering it is mandated by Canada's human rights laws. Reflecting this authority, 301 civil society organizations called for HRCs to “strengthen their official advisory role” in governments’ responses to COVID-19 (Amnesty International Canada, 2020). Analyzing these human rights authorities’ advocacy, therefore, can elucidate crucial human rights issues and obligations during this unprecedented public health emergency, and can clarify important dimensions of a human rights approach to COVID-19 response.

## Data and Methodology

The basis for analyzing Canadian HRCs’ advocacy for human rights during the COVID-19 crisis is an original dataset and content analysis (see Weber, 1990) covering all official HRC statements regarding COVID-19 and human rights until the time of writing (April 20, 2020). Statements were hand coded for the presence of particular concepts related to human rights issues and obligations (see the Appendix, available online). While HRCs may use other forms of communication (such as social media and comments to the press), these official statements published on their websites constitute the core of their human rights advocacy during the COVID-19 crisis.

## Analysis

**Figure 1.**
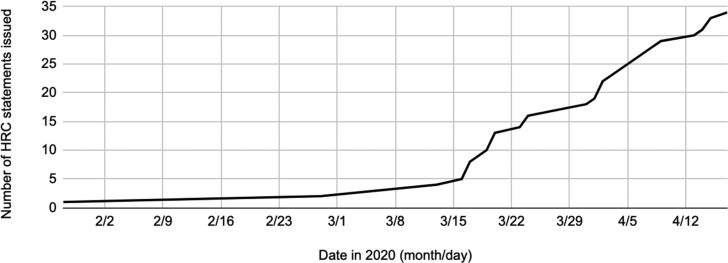
Total number of COVID-19 related statements by human rights commissions over time.

The 13 federal, provincial and territorial HRCs made a total of 34 statements. There were two statements in January and February, and statements became more frequent starting in mid-March (Figure 1). The number of statements issued by each HRC varied considerably, with Quebec's and Ontario's HRCs being the most active (Table 1). There also were qualitative differences in statement length, depth and form. Some were a few paragraphs of general recommendations; others were lengthier policy guidelines or a series of questions and answers that clarified specific human rights issues and obligations relating to COVID-19.

### The Targets of Human Rights Commissions’ Advocacy

**Figure 2.**
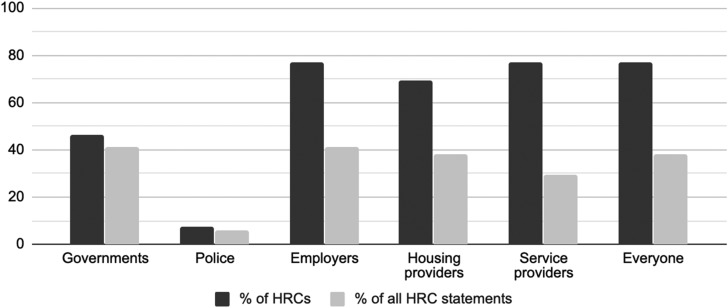
Targets of human rights commissions’ advocacy.

Some HRC statements were open-ended, but most targeted particular actors with human rights responsibilities during the COVID-19 crisis (Figure 2). These targets were consistent with HRCs’ mandates for advising governments; overseeing rights related to employment, housing and services; and educating the public on human rights. HRCs rather evenly targeted employers, housing and service providers, and people more generally. Fewer HRCs targeted governments, but the six that did released many statements on governments’ human rights obligations during COVID-19 response. Quebec's HRC uniquely targeted police and their enforcement of COVID-19 related restrictions (see, for example, CDPDJ, 2020). HRCs’ advocacy shows that a wide range of actors bear human rights responsibilities during the COVID-19 crisis.

### Justifications for Human Rights Obligations

**Figure 3.**
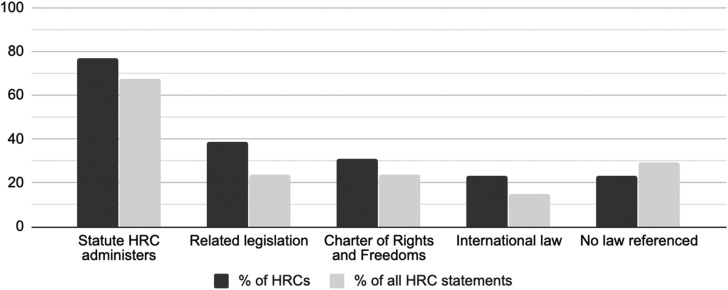
Human rights commissions’ references to human rights law.

HRCs used both legalistic and moralistic justifications (see Sharp, 2018) for respecting human rights during the COVID-19 crisis. The vast majority of HRCs advocated for human rights obligations based on human rights law (Figure 3). HRCs most frequently referenced the human rights legislation they administer, and some provincial HRCs occasionally cited related provincial legislation on employment or housing. Reflecting how HRCs do not confine their advocacy to their narrow legal mandates, several HRCs noted obligations under Canada's Charter of Rights and Freedoms and international human rights law. The Canadian Human Rights Commission (CHRC) stood out for solely relying on moralistic justifications for human rights obligations; for example, it argued that “nobody should be left behind” to justify protecting prisoners’ health and human rights during the pandemic (CHRC, 2020). This variation across HRCs shows the diversity of potential justifications for human rights obligations during the COVID-19 crisis.

### Human Rights Obligations during the COVID-19 Crisis

**Figure 4.**
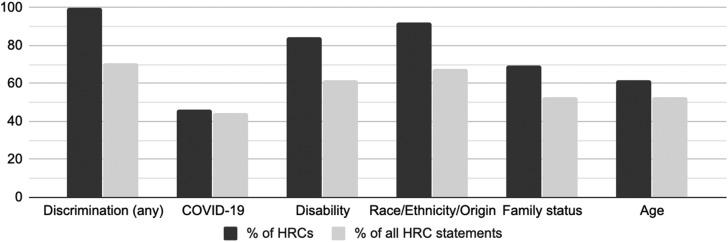
Human rights commissions’ advocacy against discrimination, including protected grounds most commonly referenced.

Given HRCs’ mandate to administer antidiscrimination-focused human rights laws, it is unsurprising that all HRCs, and the vast majority of their statements, explicitly advocated against discrimination in the context of COVID-19 and addressed specific protected grounds (Figure 4). The first HRC statements in January and February (see, for example, OHRC, 2020) responded to reports of discrimination against members of East Asian/Chinese communities and condemned discrimination based on race, ethnicity and national origin.

From mid-March onward, as the coronavirus spread and response measures intensified, statements also commonly advocated against discrimination based on disability, family status and age. Six HRCs argued COVID-19 (real or perceived) could be considered a disability with antidiscrimination protections. HRCs highlighted the prohibition of discrimination based on family status and the duty to accommodate the impacts of COVID-19 on individuals’ work and care responsibilities. Discussions of age discrimination generally focused on the vulnerability of the elderly, particularly in long-term care homes.

Figure 4 depicts the most frequently condemned forms of discrimination, but HRC statements also discussed how the detrimental impacts of COVID-19 on health, social cohesion, economic inequality, and other areas would increase the vulnerabilities of various other groups, including children, women, Indigenous peoples, homeless people, prisoners and refugees. HRCs thus advocated for their targets (Figure 2) to consider wide-ranging and multifaceted human rights obligations in their policies and practices during the COVID-19 crisis.

### Balancing Human Rights and Other Priorities

**Figure 5.**
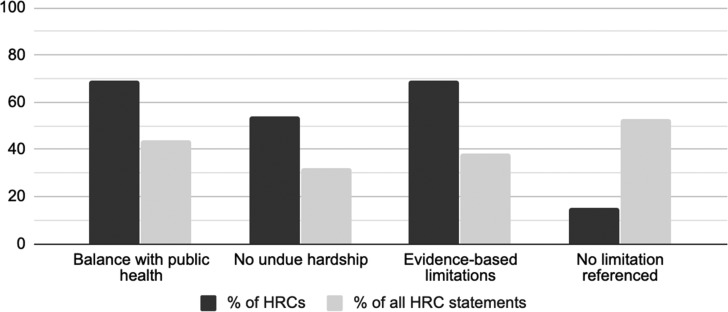
Human rights commissions’ discussion of limitations on human rights protections.

A narrow majority of HRC statements did not discuss any conditions for limiting human rights (Figure 5). Nine HRCs, however, referenced the need to balance human rights with public health and safety, and many advocated for an approach based on evidence and the latest public health guidance, rather than misinformation and stereotypes regarding COVID-19.

Seven HRCs noted that the duty to accommodate protected grounds (such as disability, family status and age) in areas such as employment, housing and services only extends up to the point of undue hardship (based on costs or health and safety concerns) for those making accommodations. Employers, for instance, should accommodate employees’ absenteeism or additional care responsibilities during quarantine or self-isolation orders, unless employers can prove that accommodations (such as flexible telework options) would involve costs or health and safety concerns amounting to undue hardship (see, for example, OHRC, 2020).

Further emphasizing the importance of evidence for human rights accountability during the crisis, HRCs targeting governments repeatedly stressed governments’ responsibilities for monitoring and reporting on the human rights dimensions of COVID-19 response. HRCs, for instance, advocated that governments should factor protected grounds, such as a race, age and disability, into their data collection, and governments should increase human rights oversight by consulting with relevant experts, stakeholders and representatives of vulnerable groups.

## Conclusion

Federal, provincial and territorial HRCs, based on their institutional authority for protecting and promoting human rights, have been strong advocates for upholding human rights protections during the COVID-19 public health emergency. Aggregating and comparing their advocacy statements reveals how far-reaching and multidimensional a human rights approach to COVID-19 response could be. Given the diversity of rights and responsibilities discussed by the various HRCs, there is room for debate regarding whether and how to reconcile the imperatives of responding to the COVID-19 emergency and protecting human rights. The broad point of consensus among all HRCs, however, is that this unprecedented crisis requires that governments and other societal actors consider threats to not only health and lives, but also a wide range of human rights.

## Note

1 Jurisdictions may have a human rights commission and/or tribunal. Tribunals, if applicable, resolve human rights complaints, with no advocacy-related mandate. Nunavut, exceptionally, has a tribunal but no HRC.

## Supplementary material

For supplementary material accompanying this paper visit https://doi.org/10.1017/S0008423920000438. click here to view supplementary material
