# Supplementary figures and images for: A Human Rights Approach to Emergency Response? The Advocacy of Canada's Human Rights Commissions during the COVID-19 Crisis
Source: Can J Polit Sci. 2020 May 6:1–7. doi: 10.1017/S0008423920000438 (PMC7248589; doi:10.1017/S0008423920000438)

**Appendix**

**Data on Human Rights Commissions and Conceptual Categories**

**
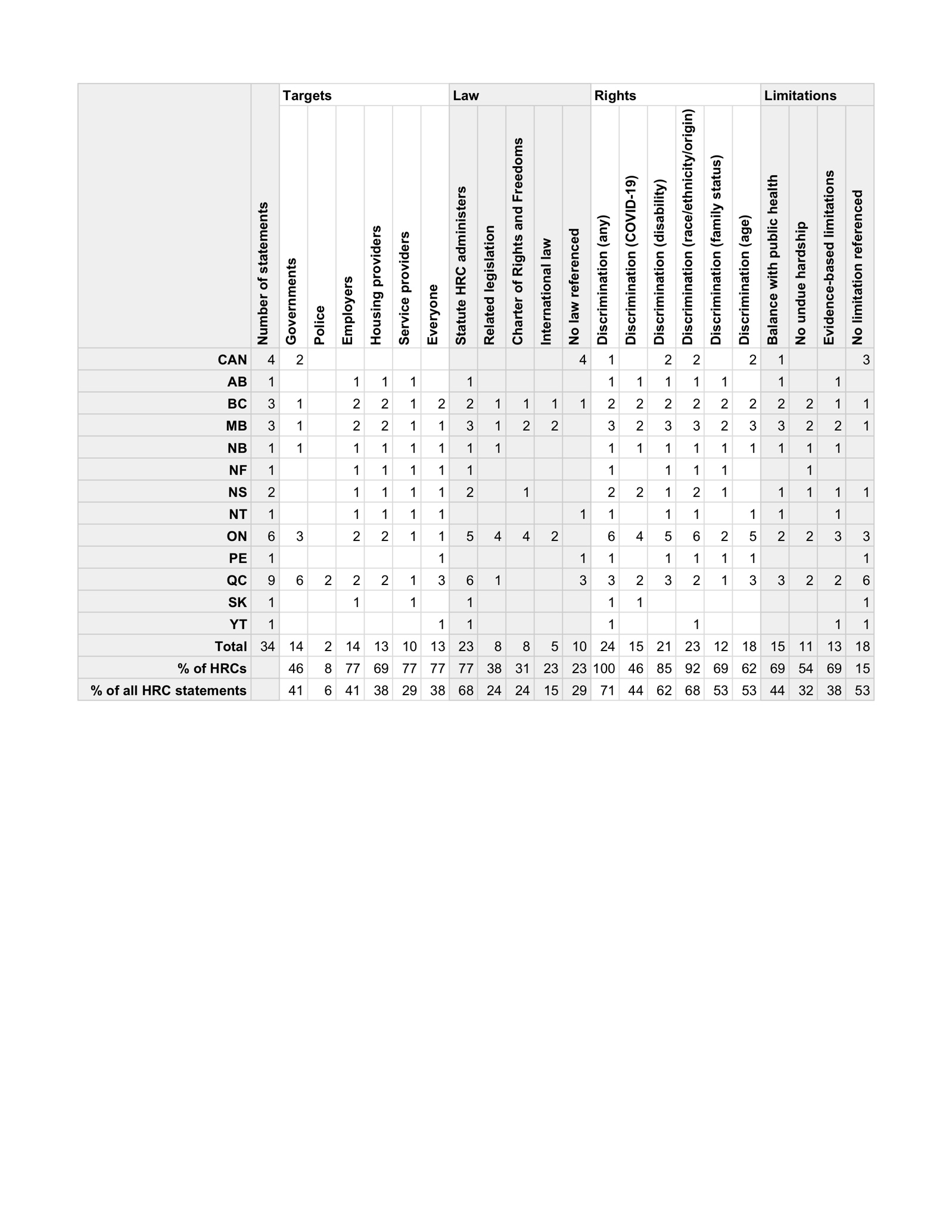
**

Supplement: Supplementary file 1 [file S0008423920000438sup001.docx]
